# Analysis and Design of a Speed and Position System for Maglev Vehicles

**DOI:** 10.3390/s120708526

**Published:** 2012-06-25

**Authors:** Chunhui Dai, Fengshan Dou, Xianglei Song, Zhiqiang Long

**Affiliations:** College of Mechatronics Engineering and Automation, National University of Defense Technology, ChangSha 410073, HuNan, China; E-Mails: daichunhui1984@hotmail.com (C.D.); dfs5460@126.com (F.D.); xiangleisong@126.com (X.S.)

**Keywords:** maglev vehicle, speed and position detection, coils, loop-cable, 8-word coils

## Abstract

This paper mainly researches one method of speed and location detection for maglev vehicles. As the maglev train doesn't have any physical contact with the rails, it has to use non-contact measuring methods. The technology based on the inductive loop-cable could fulfill the requirement by using an on-board antenna which could detect the alternating magnetic field produced by the loop-cable on rails. This paper introduces the structure of a speed and position system, and analyses the electromagnetic field produced by the loop-cable. The equivalent model of the loop-cable is given and the most suitable component of the magnetic flux density is selected. Then the paper also compares the alternating current (AC) resistance and the quality factor between two kinds of coils which the antenna is composed of. The effect of the rails to the signal receiving is also researched and then the structure of the coils is improved. Finally, considering the common-mode interference, 8-word coils are designed and analyzed.

## 1. Introduction

With the acceleration of urbanization, the density of urban rail transportation is increasing, and people are demanding much more speed, safety and comfort. The maglev vehicle is a new generation of transport, which uses electromagnetic force to achieve non-contact support and guidance, and uses a linear motor as the traction engine. It has many advantages, such as high speed, adaptability to the terrain, flexible with the route line selection, safety and environmental protection [1–4].

In Japan, the HSST (High Speed Surface Transport) is one of the representative maglev trains. It depends on a linear induction motor based on a short stator to achieve traction. To determine the position of the train, it uses the loop-cable technology and can get an accuracy of 10 centimeters. The first commercial maglev train line in China (SMT) that connects Longyang road with the Pudong International airport has been operating for about ten years now. Its highest speed is 431 km/h and it uses a linear synchronous motion actuator to achieve traction. In its synchronous traction system, SMT mainly uses two methods to detect the position of the motor's secondary. One method is to detect the counter electromotive force. The other is achieved by an inductance type transducer which can detect the alveolar structure of the track and can achieve an accuracy of one centimeter. The American corporation General Atomics (GA) also performs research on maglev train technology. It's system has a Halbach structure and also adopts a linear synchronous motion actuator like SMT. The difference is that its position detection system employs a laser reflective sensor mounted on the vehicle and optical tape on the Litz track. It can achieve an accuracy of 2 centimeters [5–8]. A detailed comparison of the different methods is shown in Table 1.

Mid-low speed maglev trains use the linear asynchronous traction method. The linear motor primary is installed at the bottom of train bogie, while the rail is the secondary. The ability to effectively obtain accurate speed information relates to the traction efficiency of the motor. In addition, accurate and timely access to the position and speed of the train, real-time transmission of train operation to the ground control center and receiving commands from the control center are the basis and guarantee of reliable operation.

This paper focuses on the method based on the inductive loop-cable. In terms of increasing the quality factor, the receiving antenna coils are designed, which lays a theoretical and experimental basis for the engineering applications of inductive loop-cable on the maglev vehicle [9–11].

## 2. System Structure Design and Principle Introduction

**Figure 1.**
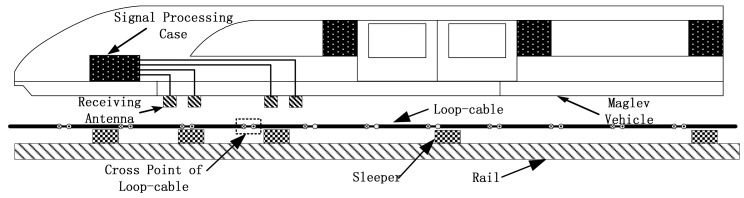
A sketch of the system based on loop-cable and maglev vehicle.

**Figure 2.**
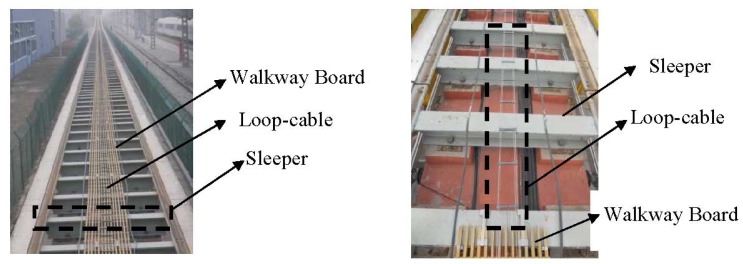
Loop-cable and sleeper.

Speed and position detection based on the inductive loop-cable applies the electromagnetic induction principle to detect the speed and position of the train. Shown in Figures 1 and 2, first of all, the loop-cable with fixed shape is laid along the rails, and the high-frequency alternating current also called the carrier current is injected into it. Meanwhile an alternating magnetic field with the same frequency is produced. As the current of the adjacent loop-cable rings runs in the opposite direction, the adjacent rings would produce the magnetic field with the opposite direction. Secondly, the receiving antenna composed of several sets of coils is installed at the bottom of the train and above the loop-cable. According to Faraday's law of electromagnetic induction, an inductive electromotive force is produced between both ends of the coils. When the train runs along the rails, the speed and position information could be determined by the amplitude and phase change of the inductive electromotive force. In the following sections, the principles of the proposed speed and position measurement method will be introduced in detail.

As the high-frequency alternating current is injected into the loop-cable on rails, an alternating magnetic field will be produced. If the direction and strength of the magnetic flux density change periodically, the magnetic flux of the receiving antenna will also change periodically. However, the amplitude of the receiving magnetic flux only changes when the train is running. The antenna uses the parallel resonance structure, and the modulated output voltage carries the position information.

**Figure 3.**
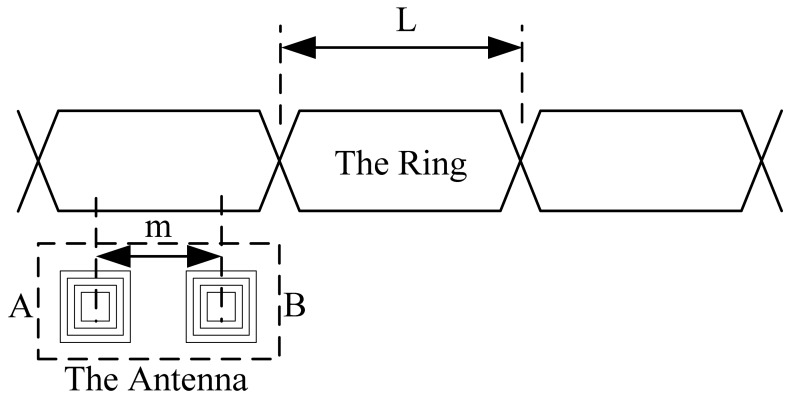
The relative position between the coils and the ring as the two coils produce voltage with the same phase.

**Figure 4.**
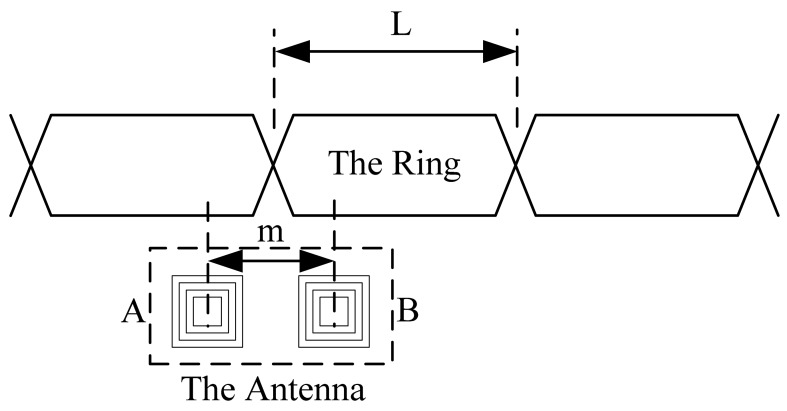
The relative position between the coils and the ring as the two coils produce voltage with the opposite phase.

The system based on the inductive loop-cable uses two receiving coils as the antenna. The center distance of the two coils is half of the ring. It is shown in Figures 3 and 4 that *m* = ½ *L*. The coil A and coil B are independent, using the same structure, the number of turns and wind direction, so ideally, the sensitivity and impendence characteristics of two coils are the same.

**Figure 5.**
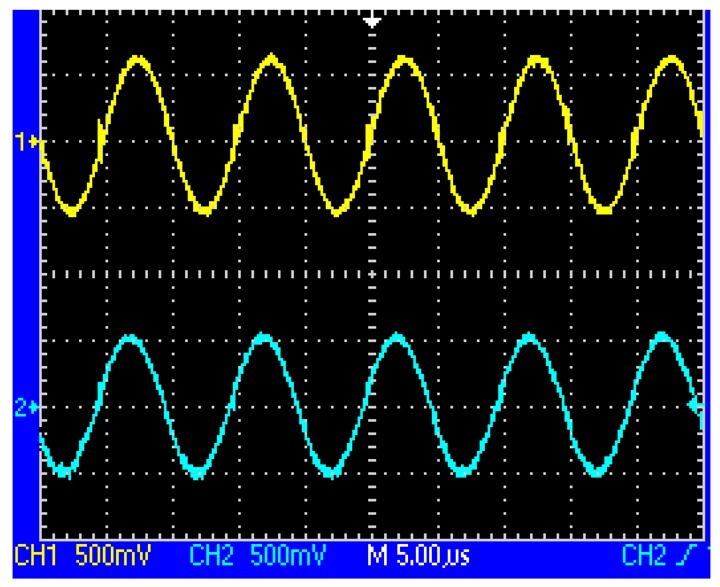
Resonant outputs of the coils with the same phase.

**Figure 6.**
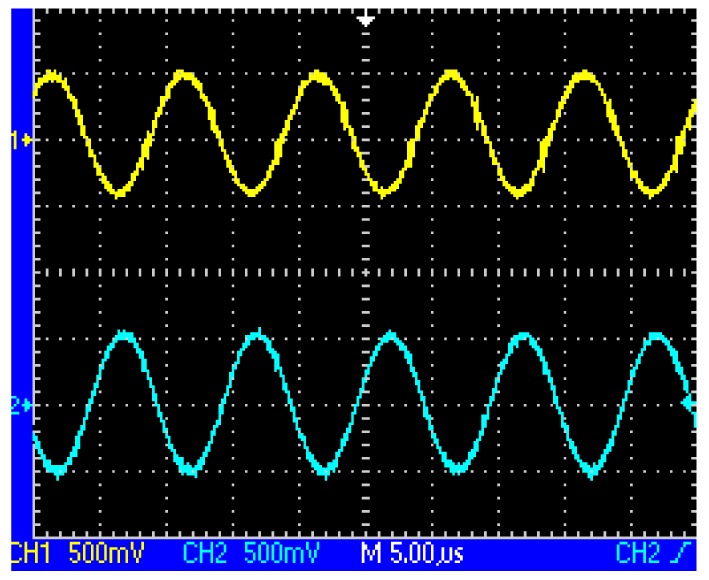
Resonant outputs of the coils with the opposite phases.

When the two coils are located above the same loop-cable ring shown in Figure 3, the phase of the receiving voltage is nearly the same. But when they are located above the adjacent loop-cable rings shown in Figure 4, the phase difference of the receiving signals is nearly 180 degrees. The output signals of the two coils which are processed by a parallel resonant circuit are shown as Figures 5 and 6. As the antenna composed of the two coils moves in the direction of the loop-cable, the speed and position information could be measured by comparing the phase changes of the output voltages.

**Figure 7.**
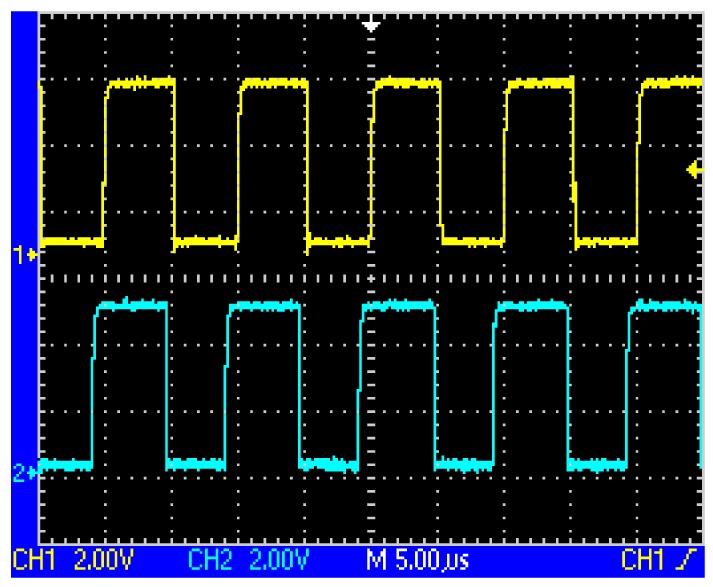
The respective outputs after dealing with the waves shown in Figure 5 using zero crossing detector.

**Figure 8.**
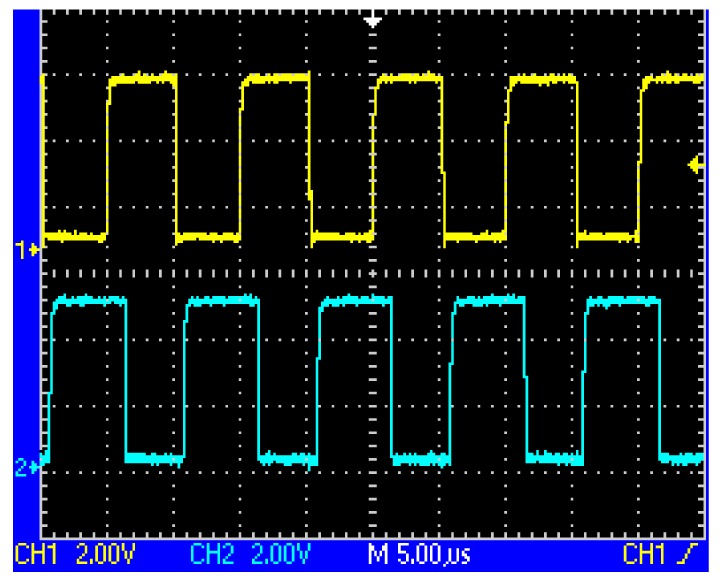
The respective outputs after dealing with the waves shown in Figure 6 using zero crossing detector.

After the following process includes voltage tracking, linear amplification, band pass filtering, and zero crossing treatment, the square waves as shown in Figures 7 and 8 could be obtained, whose phases change alternately. Still further, the exclusive or (XOR) processing is employed, so a set of pulse signals could be obtained, which is also called speed pulse. For example, in the case of Figure 3 logic ‘0’ is produced while in the case of Figure 4 logic ‘1’ is produced. The period of the pulse signal is T, so the relationship between pulse period and train speed is given by:

(1) $$v=\frac{L}{T}$$

**Figure 9.**
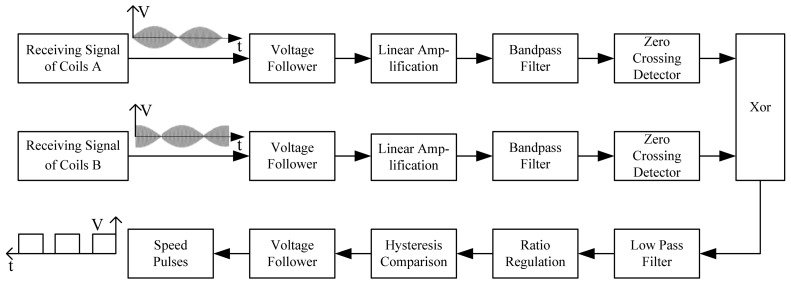
Structural drawing of signal processing.

It should be pointed out that the maglev train needs the upward force of the suspension provided by the electromagnet, and a variety of high-power devices on board working simultaneously is bound to generate electromagnetic fields in the surrounding environment, so there is severe electromagnetic interference around the antenna. Although the resonant circuit has the function of selective filtering of certain frequencies and anti-common-mode interference, an active filter circuit is needed to perform a progressive filtering and improve the signal to noise ratio. This is shown in Figure 9 below.

## 3. The Electromagnetic Field Analysis of Loop-Cable

**Figure 10.**
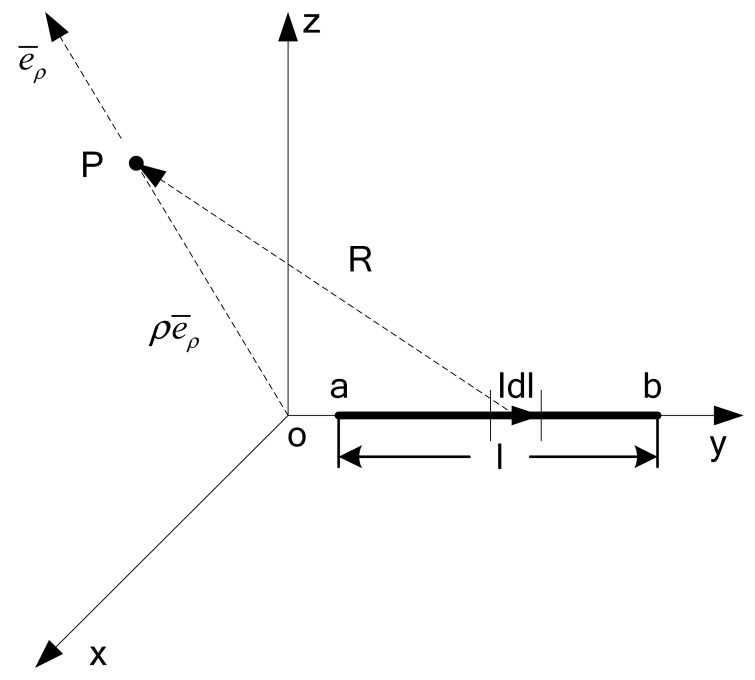
The magnetic flux density of finite current-carrying conductor.

Firstly, the magnetic flux density of limited long current-carrying wire is calculated, while the point P is generated arbitrarily. This is shown in Figure 10. As *Idl̅* = *Idze̅_y_* and *R̅* = *ρe̅_ρ_* − *ye̅_y_* it could be obtained that:

(2) $$Id\bar{l}\times \bar{R}=I\rho dy{\bar{e}}_{\phi }$$

According to the Biot-Savart law, the magnetic flux density of the point P in Figure 10 is as follows:

(3) $$\begin{array}{l} \bar{B}=\frac{\mu_{0}I\rho }{4\pi }\int_{a}^{b}\frac{dz}{{\left[\rho^{2}+b^{2}\right]}^{3/2}}{\bar{e}}_{\phi }\\ \;=\frac{\mu_{0}I}{4\pi \rho }[\frac{b}{\sqrt{\rho^{2}+b^{2}}}-\frac{a}{\sqrt{\rho^{2}+a^{2}}}]{\bar{e}}_{\phi } \end{array}$$

**Figure 11.**
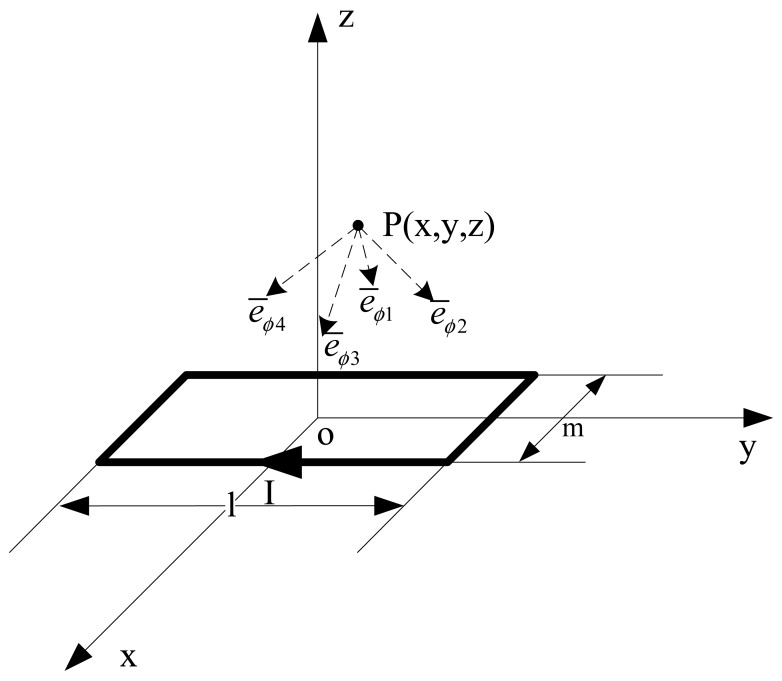
The magnetic flux density of square current-carrying coil.

Secondly, we could calculate the flux density at any point P outside the square coil with current I, which is shown as Figure 11.

The origin of the space coordinates is at the center point of the geometric symmetry of the square coil, se we can get the formula:

(4) $$\bar{B}=\frac{\mu_{0}I}{4\pi \sqrt{z^{2}+\frac{m^{2}}{4}}}[\frac{\alpha_{1}}{\sqrt{z^{2}+\frac{m^{2}}{4}+{\alpha_{1}}^{2}}}+\frac{\alpha_{2}}{\sqrt{z^{2}+\frac{m^{2}}{4}+{\alpha_{2}}^{2}}}]{\bar{e}}_{\phi 1}+\frac{\mu_{0}I}{4\pi \sqrt{z^{2}+\frac{l^{2}}{4}}}[\frac{\beta_{1}}{\sqrt{z^{2}+\frac{l^{2}}{4}+{\beta_{1}}^{2}}}+\frac{\beta_{2}}{\sqrt{z^{2}+\frac{l^{2}}{4}+{\beta_{2}}^{2}}}]{\bar{e}}_{\phi 2}+\frac{\mu_{0}I}{4\pi \sqrt{z^{2}+\frac{m^{2}}{4}}}[\frac{\alpha_{1}}{\sqrt{z^{2}+\frac{m^{2}}{4}+{\alpha_{1}}^{2}}}+\frac{\alpha_{2}}{\sqrt{z^{2}+\frac{m^{2}}{4}+{\alpha_{2}}^{2}}}]{\bar{e}}_{\phi 2}+\frac{\mu_{0}I}{4\pi \sqrt{z^{2}+\frac{l^{2}}{4}}}[\frac{\beta_{1}}{\sqrt{z^{2}+\frac{l^{2}}{4}+{\beta_{1}}^{2}}}+\frac{\beta_{2}}{\sqrt{z^{2}+\frac{l^{2}}{4}+{\beta_{2}}^{2}}}]{\bar{e}}_{\phi 4}$$

where $\alpha_{1}=\frac{l}{2}-y$, $\alpha_{2}=\frac{l}{2}+y$, $\beta_{1}=\frac{l}{2}-x$, $\beta_{2}=\frac{l}{2}+x$; *ē_φ_*_1_, *ē_φ_*_2_, *ē_φ_*_3_ and *ē_φ_*_4_ stand for the directions of flux density due to the four lines of the square coil separately.

**Figure 12.**
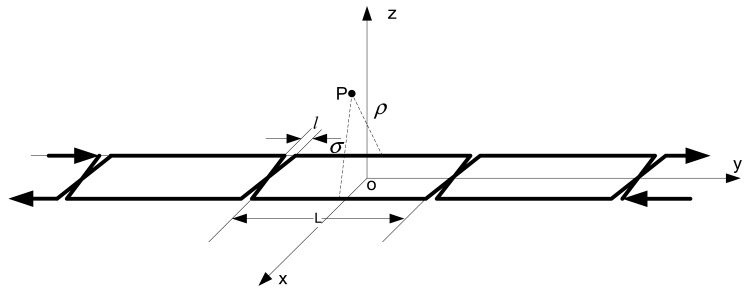
Model of loop-cable with current.

**Figure 13.**
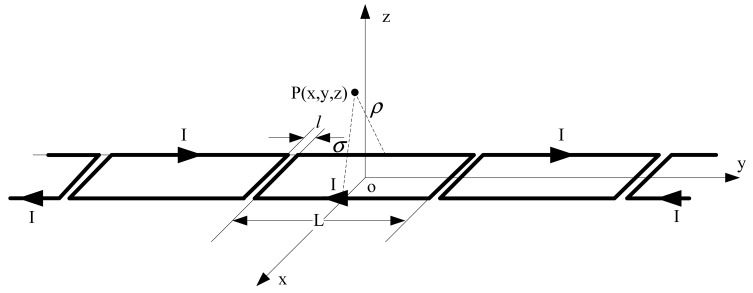
Equivalent model of loop-cable with current.

Figure 12 shows the loop-cable which is laid on the rails. In fact *l* ≪ *L*, so the model of the loop-cable could be equivalent to a number of square coils arranged by turns. Suppose that the square coils are injected with the current *I* and the cross-cycle of the loop-cable is long of L. The surface of every square coil is in the xoy plane of the space coordinate system. This is shown as Figure 13.

The magnetic flux density of any point P outside the loop-cable is the superimposition of every square coil with current. Due to the cyclical layout of the square coils, the magnetic flux density is also regularly distributed along the y-axis. The flux density of the point P could be decomposed into three components in the x, y and z directions:

(5) $${\bar{B}}_{p}=f(x,y,z)I{\vec{e}}_{x}+g(x,y,z)I{\vec{e}}_{y}+h(x,y,z)I{\vec{e}}_{z}$$

**Figure 14.**
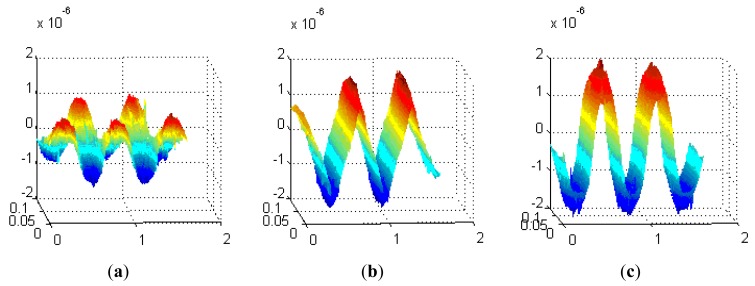
The magnetic flux density components of x, y and z direction (**a, b** and **c**).

The detection height from the loop-cable is about ten centimeters, and the corresponding decomposed magnetic flux density curves are shown as Figure 14(a–c). The z-axis unit is Tesla (T), while the y-axis and x-axis units are meters (M). We may select one of the three direction components as the detection object and use it to calculate the speed and position of the train.

From what are shown in Figure 14, the x direction component of magnetic flux density is relatively small, and therefore it is not suitable as the measured component. As the second section introduces, the speed and position detection system requires that the antenna be sensitive to the direction change of the magnetic field. From what is seen in Figure 14(b), the y direction component of magnetic flux density changes direction at the center position of each equivalent ring while the z direction component of magnetic flux changes at the crossover position. This kind of difference has essentially no effect on the speed and position detection.

As we know the speed pulse jumps at the cross point, but in fact the flux of the coils may be very small nearby, so there is a shadow area. To decrease the shadow area, it is desirable that the change rate of the flux density be as big as possible. Comparing the z and y direction components, the change rate and magnetic flux density of the z-direction one is bigger, so it is more suitable as the measured component than the y-direction component. Meanwhile the orientation of the antenna could also be determined, and it is parallel to the plane of the loop-cable.

## 4. Design of the Receiving Antenna

### 4.1. Basic Working Principles of the Coils

According the law of electromagnetic induction, the inductive electromotive force of a single-turn coil is as follows:

(6) $$\xi =-\int_{\text{S}}\frac{\partial \bar{B}}{\partial t}\cdot d\bar{S}+\oint_{l}(\bar{v}\times \bar{B})•d\bar{l}$$

where *B̄* is the magnetic flux density produced by the loop-cable; *v̄* is speed that the antenna moves relatively to the loop-cable, which is nearly equal to the speed of the train.

The first term on the left of Equation (6) is the induced electromotive force while the second one is the motional electromotive force. Considering that the speed of a mid-low speed maglev train is usually less than 120 km/h, *i.e.*, v ≤ 33 m/s and the frequency of the carrying current in the loop-cable is very high, the motional electromotive force is much smaller than the induced electromotive force, so it could be ignored (the motional electromotive force is also not considered in the following analysis). Usually the receiving coils use the N turns winding mode, so according to Equation (6), the induction electromotive force between two ends of the coils is:

(7) $$\xi =-N\int_{\text{S}}\frac{\partial \bar{B}}{\partial t}\cdot d\bar{S}$$

**Figure 15.**
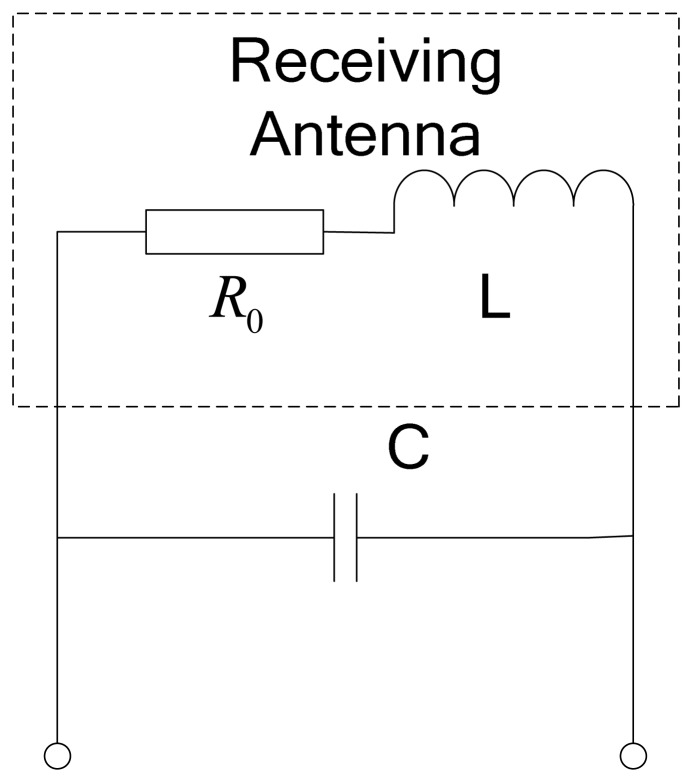
The basic parallel resonant circuit.

In most receiving antenna circuits, to ensure the output voltage of the coils is as large as possible, usually the parallel resonant mode is adopted, as shown in Figure 15. It also has the function that it only receives the signal at a particular frequency, and effectively inhibits signals at other frequencies, *i.e.*, is has frequency selection. The loss of the coils is regarded as the series resistance *R*_0_. Supposing the frequency of the input signal is *ω*_0_, the appropriate capacitor should satisfy *LC* ≈ 1/*ω*^2^ in the reference to impedance matching theory. Only in this manner the equivalent maximum impendence could be obtained.

The character of frequency selection is related with the circuit quality factor. The quality factor is calculated as follows:

(8) $$Q=\frac{\omega_{0}L}{R_{0}}$$

where *R*_0_ is also called the AC impendence of the coils, *L* is the inductance, *ω*_0_ is the resonant frequency, which is equal to the carrier frequency of loop-cable. Usually as the quality factor increases, the character of frequency selection gets better.

In fact, the voltage and current in the parallel uniform transmission line are generally the superposition of the incident wave and reflected wave. To avoid reflection, generally the terminal resistance is added. But the loop-cable that this paper researches has a special periodic crossover structure; it is difficult to find a suitable impedance matching means to eliminate the signal reflection caused by long distance transmission. Therefore, in order to improve the signal transmission quality, it is necessary that the length of the loop-cable be less than one eighth of the signal wavelength, so under the condition of an actual loop-cable lenght of five hundred meters, the frequency of the carrier current is better at less than 100 kHz [12,13]. Different kinds of coils have different impedance characteristics, so the design of coils will be researched in the following section.

### 4.2. Design and Analysis of Two Different Kinds of Coils

**Figure 16.**
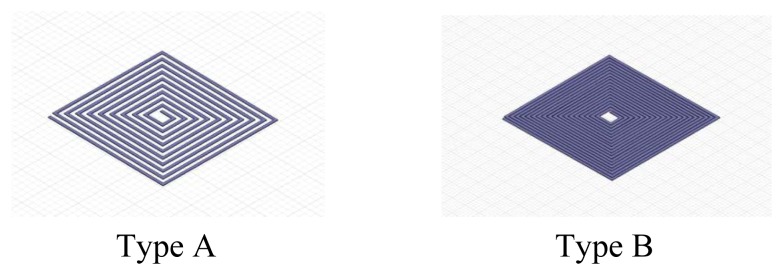
The coils made of printed circuit board PCB.

Firstly the coils A and B shown in Figure 16 are considered. They are made of printed circuit board (PCB). The coils wind around the center, and are located in the same plane. Their sizes are 10 cm × 10 cm. A has a number of turns of 20, while B has 40 turns [14–16]. The fabrication of these two types of coils is simple; their consistency is nice and the distributed parameters are relatively fixed. As we all know, for the isotropic material, if the magnetic field is generated by a current loop, the ratio of the flux through the loop to its inner current is the inductance of the coil loop.

**Figure 17.**
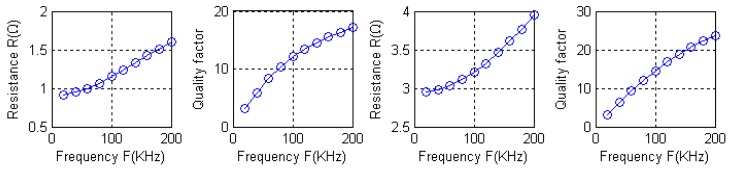
AC impendence and quality factor on the function of frequency.

Intuitively, due to the fact both type A and type B coils using the coil nested winding method, the magnetic field generated by the external coil loop and the magnetic field generated by the internal coil loop cancel each other out, making the coils' inductance and quality factor relatively small. Figure 17 shows the curves of AC impendence and quality factor as a function of frequency. When driven by a signal with a frequency of 100 kHz, the measured quality factors are 12.08 and 14.52.

**Figure 18.**
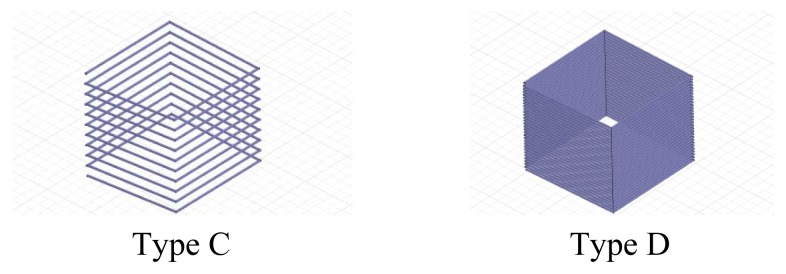
The coils made of enamel-covered wire.

If the coil winding uses the method shown in Figure 18, the low quality factor problem might be overcome. The type C and type D coils shown in the figure use enamel-covered wire with a diameter of 0.5 mm, and their sizes are 10 cm × 10 cm. Type C has 20 turns and Type D has 40 turns.

**Figure 19.**
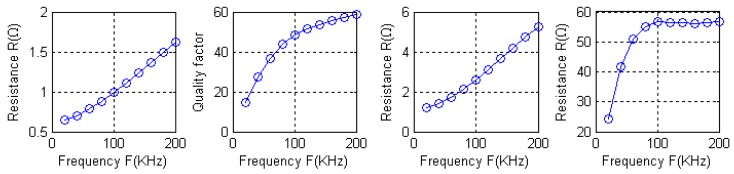
AC impendence and quality factor on the function of frequency.

Figure 19 shows the curves of the quality factor as a function of frequency. Also driven by a frequency of 100 kHz signal, their quality factors are 56.71 and 59.27. Although type A and type C have the same number of turns, the latter has a much larger quality factor. This is undoubtedly beneficial to signal reception. Comparing type C and type D, the turn number of the latter is twice as large as in the former. Under the condition of the same magnetic field distribution, the more the number of turns, the greater the magnetic flux of the coils and the signal receiving strength, therefore, an appropriate increase in the number of turns is beneficial for the signal reception.

In addition, Equation (8) shows that the quality factor is related with AC impedance, the inductance and the resonant angular frequency. In the case of high frequency driving, due to the appearance of skin effects, the current and electromagnetic field distribute on the surface of the conductor. As the wire diameter is greater than several depths of penetration, the current may get smaller with the increasing frequency. Even if the cross sectional area of the conductor is relatively large, most of it is not used and the actual current-carrying cross sectional area is reduced. Therefore, AC resistance is different from DC resistance and as the frequency increases, the resistance will become larger, so the quality as a function of frequency is not linear. Figure 19 shows the result. Especially, the quality factor shows almost no change as the frequency changes between 100 kHz and 200 kHz.

### 4.3. Analysis about the Effect of Sleeper

The above analysis about the electromagnetic field is based on the loop-cable being located in a vacuum environment. However, there is always other material nearby, and then the alternating magnetic field produced by the loop-cable might be affected. The main aspects that need to be considered are magnetization and eddy current effects [17–19].

Different materials have different permeability, which may change the magnetic resistance of the spatial magnetic field. For isotropic materials, the magnetic field strength and magnetization are related as follows:

(9) $$M=\chi_{m}H$$

where M is a vector representing the magnetization, *χ_m_* is the magnetic susceptibility and H is a vector representing the magnetic field strength. It could be obtained that:

(10) $$B=\mu_{0}[1+\chi_{m}]H=\mu_{0}\mu_{r}H=\mu H$$

where *μ*_0_ is vacuum permeability, *μ_r_* is material relative permeability and *μ* is material permeability.

For paramagnetic and anti-magnetic materials, the relative permeability is very small, so it could be supposed that *μ_r_* = 1. But as for ferromagnets in a flux density 1T, the relative permeability may be as high as 5000. When a ferromagnet is placed in the magnetic field, magnetic dipoles within the ferromagnet are arranged along the direction of the field, and so its magnetic reluctance is very small. The result is that the spatial magnetic field increases.

Near the loop-cable and the on-board antenna, bulk metal often exists, including metal sleepers laid on the loop-cable below, the metal bogie where the antenna is installed, and the linear motor stator. Due to the small resistance of bulk metal, the eddy current could often reach a very large intensity. Because of the different reasons for the changes of magnetic flux, there are two kinds of cases. One is in a constant magnetic field where the change of magnetic flux is due to the conductor motion, so an inductive electromotive force is caused which is called motional electromotive force. The other one is where the conductor is fixed, and the magnetic field is changing, which will also result in an inductive electromotive force called induced electromotive force. Although both are caused by the magnetic flux changes, their physical essence is not the same. The former electromotive force is provided by the Lorentz force, while the latter one is provided by the eddy current field force. The loop-cable is injected with a high-frequency carrier current, which results in an alternating magnetic field, so in this paper, the eddy current is due to the latter.

**Figure 20.**
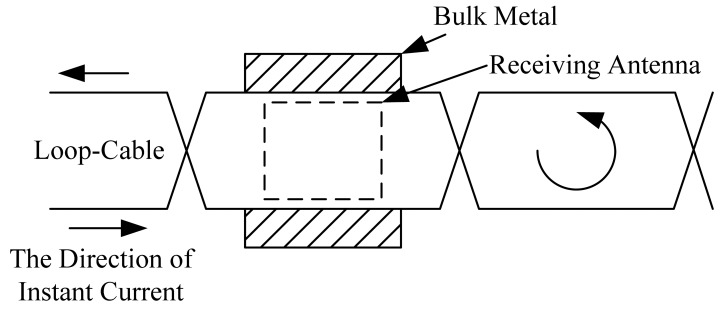
The relative position of bulk metal and loop-cable.

**Figure 21.**
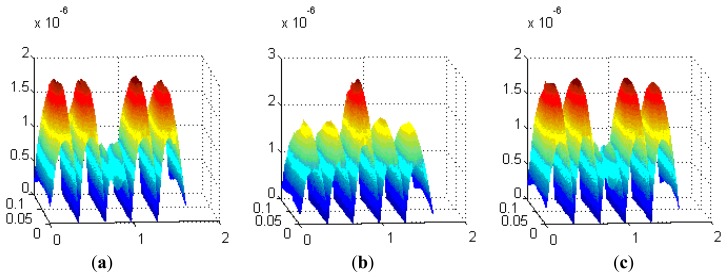
The spatial magnetic field affected by different materials.

For any kind of material, both magnetization and eddy current effects exist. Compared with the vacuum magnetic field distribution, the first major impact is an enhancement of the spatial magnetic field, while the second is to weaken it. In addition, the eddy current effect is related with the frequency of the magnetic field. Under the condition where the loop-cable is injected with the same AC current, three kinds of metal (aluminum, iron and stainless steel) are placed separately in the position shown in Figure 20. The spatial distribution of the z direction component of the magnetic field is shown in Figure 21, which is a simulation result produced by Ansoft.

The relative permeability of aluminum approximates 1, so it has little effect on the distribution of the reluctance of the magnetic field around the loop-cable. However its conductivity is as high as 3.8 × 10^7^ S·m^−1^, and as it is close to the loop-cable, an eddy current will be produced which could counteract the magnetic field. This result is shown in Figure 21(a). Similarly, for stainless steel, its electrical conductivity is high, but its relative permeability is very small, and so the spatial distribution of the magnetic field also gets weaker, as shown in Figure 21(c). However, due to the fact that the relative permeability of iron may be as high as 5,000, it reduces the equivalent series magnetic circuit reluctance, thus increasing the spatial magnetic field produced by the loop cable. This is shown in Figure 21(b).

**Figure 22.**
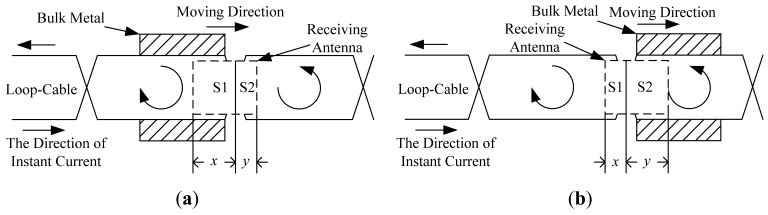
The relative position of receiving coils and loop-cable considering the bulk metal or sleeper.

As the receiving antenna is close to the crossing point of loop-cable, due to the opposite directions on both sides of the alternating magnetic flux density, the magnetic flux of the coils cancels out, which makes the inductive electromotive force nearly zero. With the antenna is moving, the output phase will change 180 degrees. But if a sleeper of another metal is near or just below the position shown in Figure 22, the magnetic field will be distorted. As the antenna happens to be passing, the problem arises that the direction of the output inductive electromotive force will be inverted in delay (shown in Figure 22(a)) or in advance (shown in Figure 22(b)). The accuracy of position detection is directly affected. The effect is analyzed in the following section.

The antenna in Figure 22 is divided into two receiving regions: S1 and S2, meanwhile it is assumed that the amplitude of the z-direction component of magnetic flux density within the same region is the same, i.e., *B_z_*_1_ and *B_z_*_2_, respectively. Then the flux of the coils is as follows:

(11) $$\psi =N(\phi_{1}+\phi_{2})=N(B_{z1}S_{1}+B_{z2}S_{2})$$

The inductive electromotive force of the coils output could be decomposed as follows:

(12) $$U=-\frac{d\psi }{dt}=U_{1}+U_{2}=-N\frac{dB_{z1}}{dt}S_{1}-N\frac{dB_{z2}}{dt}S_{2}$$

where $U_{1}=-N\frac{dB_{z1}}{dt}S_{1}$, $U_{2}=-N\frac{dB_{z2}}{dt}S_{2}$.

In the ideal condition, as the coils' centers are just located in the crossing point of the loop-cable ring, it could be obtained that *S*_1_ = *S*_2_. The amplitudes of the z-direction magnetic field *B_z_*_1_ and *B_z_*_2_ are equal, |*B_z_*_1_| = |*B_z_*_2_|. However the directions are opposite, so the output electromotive force is nearly zero. As the receiving antenna coils' center moves across the crossing point, the phase of the output electromotive force will change by 180 degrees. The analog processing circuit shown in Figure 3 could easily produce speed pulses just by detecting the phase reversal.

First considering the coils off-center position left, as shown in Figure 22(a), the left side area is larger than the right side area. Under the condition of the absence of bulk metal, the phase of the output voltage U is determined by *U*_1_. As the coils move from left to right along the ring laying direction, the direction of the electromotive force will be inverted. However, when a piece of metal, taking stainless steel as an example, is placed in the position shown in Figure 22(a), the spatial magnetic field is affected by the eddy current, which attenuates the amplitude of the left magnetic flux density. According to the Equation (12), if in this position, *B_z_*_1_*S*_1_ = *B_z_*_2_*S*_2_ is satisfied, which leads to *U*_1_ + *U*_2_ = 0. Then the phase changes in advance here, and it results in the speed pulse deviation given by:

(13) $$\Delta e=x-\frac{d}{2}$$

where *d* is the length of the coils and it satisfies *x* + *y* = *d* shown in Figure 21; *W* is the width of the coils. Because *S*_1_ = *W*×*x*, *S*_2_ = *W*×*y* and *B_z_*_1_*S*_1_ = *B_z_*_2_*S*_2_, it could be obtained that:

(14) $$\frac{S_{1}}{S_{2}}=\frac{x}{y}=\frac{B_{z2}}{B_{z1}}=\frac{1}{\alpha }$$

Deviation on the expression of the coils length is obtained by:

(15) $$\Delta e=x-\frac{d}{2}=d(\frac{1}{1+\alpha }-\frac{1}{2})$$

**Figure 23.**
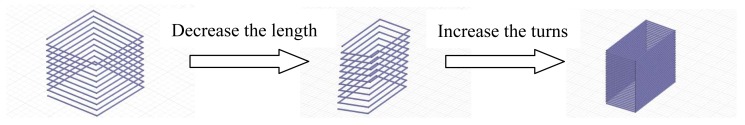
The coils change.

Similarly, as the bulk metal is moved away and the coils move to the position shown in Figure 22(b), the left side area is smaller than the right side area, and then the phase of the output voltage U is determined by *U*_2_. As the coils move from left to right along the ring, the output of the electromotive force will not be inverted. However, when a piece of metal is present, also stainless steel, the spatial magnetic field is affected by the eddy current, which attenuates the amplitude of the right magnetic flux density. There may exist one position where *B_z_*_1_*S*_1_ = *B_z_*_2_*S*_2_, that is *U*_1_ + *U*_2_ = 0. Then the phase changes in delay here, and it also results in a speed pulse deviation:

In fact, as known from Equation (15), if the length of the coils is reduced, the deviation may be reduced, while in order to ensure the signal strength is not weakened, the number of winding turns should be increased and the structure is redesigned as illustrated in Figure 23:

### 4.4. Design of 8-Word Coils

**Figure 24.**
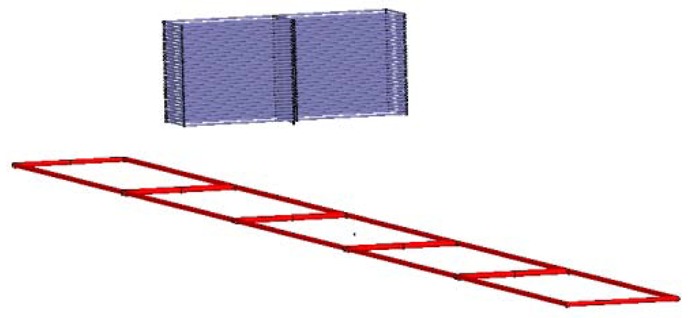
The relative position of 8-word coils and loop-cable.

**Figure 25.**
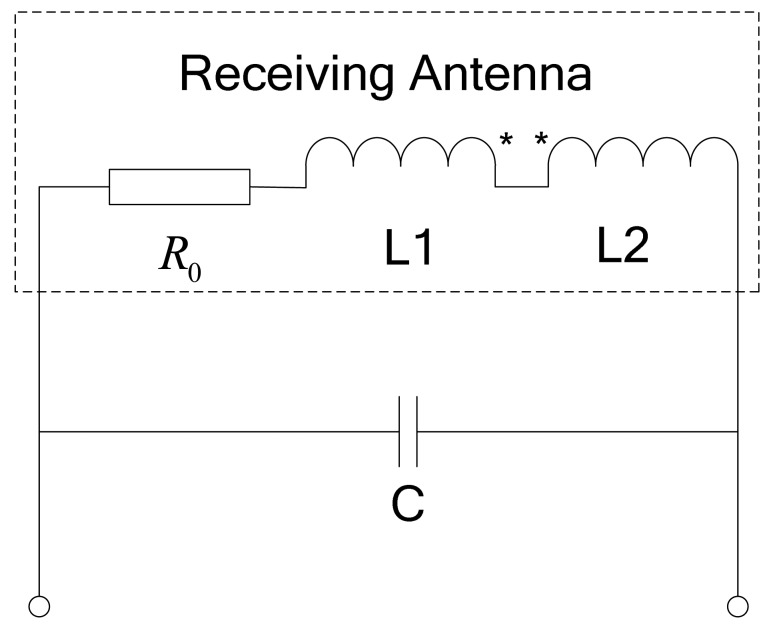
The equivalent resonant circuit of 8-word coils.

The coils described above are based on a single-coil structure. However the maglev train adopts an open linear motor and its speed is controlled by an AC motor. The current rectifier and DC to AC inverter of the AC motor have steep rising and falling edges, which may generate wide-band harmonic components, including the one with the same frequency of the carrier current. The antenna with single-coil structure is not able to distinguish between such an interference signal and the effective carrier signal. In order to eliminate this common-mode interference, the anti-interference design shown in Figure 24 is implemented and it equivalent resonant circuit is shown in Figure 25. Receiving coils L1 and its counterbalance coils L2 are installed on the same plane with their dotted terminals in series; meanwhile it is better to ensure that their parameters are the same, including the number of turns and size.

The connected coils (receiving coils and counterbalance coils) are parallel with capacitor C, which comprises the resonant circuit. As the carrier current is injected into the loop-cable, coils L1 could generate the electromotive force for it faces the ring of the loop-cable. However coils L2 could generate a very weak electromotive force as they deviate from the ring, but in terms of the interference source placed at a distance, which is approximately equal to that of coils L1 and coils L2, the inductive electromotive force generated by the two coils is nearly equal, so the differential structure due to the series of dotted terminals greatly weakens the noise strength.

## 5. Conclusions

This paper briefly describes the structure of a speed and position detection system based on a loop-cable, and analyzes the electromagnetic field produced by this loop-cable. The magnetic flux density distribution map is given for the condition of 10 cm height. The z-direction component is chosen as the measured component according to the strength and change rate of magnetic flux density, and thus the orientation of the receiving antenna is determined. This paper also designs two kinds of coil structures and compares their AC impendence and quality factors. The structure based on type C or type D is selected as the basic structure of the antenna. Considering that the sleeper or other bulk metal would affect the spatial magnetic field distribution, the size of the coils is further optimized, which could make errors smaller. Finally, considering the receiving antenna is subject to electrical interference, the 8-word coils are designed, which could effectively remove most of the common-mode interference and make the system more reliable.

## Figures and Tables

**Table 1.** Comparisons of the different measurement methods used in HSST, SMT and GA.

	**Method**	**Precision**	**Type of Traction**	**Measuring condition**
HSST	Loop-cable	10 cm	Linear asynchronous motor	Speed < 100 km/h
SMT	Alveolus detection using inductance type transducer	About 1 cm	Linear synchronous linear motor	Speed < 20 km/h
	Detecting counter potential force	*		Speed > 20 km/h
GA	Using laser sensor and optical tape	About 2 cm	Linear synchronous motor	*

* notes that it is not clear.
